# Is early time to positivity of blood culture associated with clinical prognosis in patients with *Klebsiella pneumoniae* bloodstream infection?

**DOI:** 10.1017/S0950268823000262

**Published:** 2023-02-21

**Authors:** Weiwei Hou, Tiantian Han, Guangbo Qu, Yehuan Sun, Dianyu Yang, Yan Lin

**Affiliations:** 1 Department of Laboratory Medicine, Tongji Hospital, School of Medicine, Tongji University, Shanghai 200065, China; 2 Department of Hospital Infection Control, Tongji Hospital, School of Medicine, Tongji University, Shanghai 200065, China; 3 Department of Epidemiology and Biostatistics, School of Public Health, Anhui Medical University, Hefei, 230032, Anhui Province, China

**Keywords:** Bloodstream infection, *Klebsiella pneumoniae*, prognosis, time to positivity

## Abstract

The association between time to positivity (TTP) of blood culture and the clinical prognosis of patients with *Klebsiella pneumoniae* bloodstream infection (BSI) remains unclear. A retrospective study of 148 inpatients with BSI caused by *K. pneumoniae* was performed at Shanghai Tongji Hospital, China, from October 2016–2020. The total in-hospital fatality rate was 32%. The median TTP was 11.0 (7.7–16.1) h and the optimal cutoff for prediction of in-hospital mortality was 9.4 h according to the ROC curve. Early TTP (<9.4 h) was a risk factor for in-hospital mortality by univariate analysis (OR = 2.5, 95% CI 1.2–5.0, *P* = 0.01), but not by multivariate analysis (OR = 2.7, 95% CI 1.0–7.4, *P* = 0.06). Old age, serum creatinine, white blood cells, and C-reactive protein values were risk factors for in-hospital mortality by multivariate analysis. Early TTP was not a risk factor for septic shock (OR = 1.8, 95% CI 0.6–5.1, *P* = 0.27) or ICU admission (OR = 1.0, 95% CI 1.0–1.0, *P* = 0.32). In conclusion, the in-hospital fatality rate of patients with *K. pneumoniae* BSI was relatively high and associated with an early TTP of blood cultures. However, no increased risk of mortality, septic shock or ICU admission was evident in early TTP patients.

## Introduction

*Klebsiella pneumoniae* (*K. pneumoniae*), a natural resident of the normal human faecal microbiome, can cause community and hospital-acquired infections such as pneumonia, liver abscess, bacteraemia and intraperitoneal and urinary tract infections [1]. Multidrug resistance is common in the species, ranging from 36.0% to 73.2% and showing an increasing trend [2–4]. *K. pneumoniae* bloodstream infection (BSI) is more likely to occur in patients who require relatively long-term health care, and is associated with high mortality [4], particularly if caused by carbapenem-resistant strains [5]. Therefore, appropriate indicators are needed to assess the possible prognosis for patients and inform therapy for such infections.

Time to positivity (TTP) is the time interval from incubation of a blood culture sample to a positive signal of bacterial growth, which is associated with bacterial load [6]. Previous studies have emphasised the value of TTP in the diagnosis of catheter-related BSIs [7], potential differentiation of pathogens [8] and for some species a predictor of death in patients with BSI [9] caused by various pathogens such as *Staphylococcus aureus* [10] and *Pseudomonas aeruginosa* [11]. A short TTP (<7 h) was found to be significantly associated with mortality at all time points after admission for patients with *K. pneumoniae* monomicrobial bacteremia [12], and an early TTP (≤13 h) was identified as an independent risk factor for in-hospital mortality for paediatric patients with this organism [13]. However, no direct evidence has been presented of an association between TTP and 28-day mortality for patients with *Klebsiella spp.* BSI, in general [6], and indicates the need for further studies of such a correlation, as well as the most appropriate cut-off TTP value for these patients.

As a consequence, the present study was undertaken to investigate further the correlation between TTP values of blood cultures and clinical prognosis, verify its capacity as a predictor of in-hospital mortality, and determine the most appropriate cut-off value to inform mortality among patients with *K. pneumoniae* BSI.

## Methods

### Study design and patients

A retrospective study was performed at Shanghai Tongji Hospital, China. Patients with *K. pneumoniae* monomicrobial BSI were selected from clinical and laboratory records from October 2016 to October 2020. The inclusion criteria for subjects were: inpatients with a positive blood culture for *K. pneumoniae* and with recorded TTP values. Patients were excluded if they were admitted from the ICU or emergency department and if they fulfilled any of the outcomes such as death or septic shock, before the blood culture or had polymicrobial BSIs.

### Definitions

*K. pneumoniae* BSI was defined as a positive blood culture result for the species. Antimicrobial resistance of isolates was categorised as either CRKP (resistant to carbapenems) or multi-drug resistant (MDRKP, resistant to ≥3 different classes of antibiotic agents). TTP was defined as the time interval from incubation of the blood culture sample to a positive signal, and the shortest value was recorded when multiple positive signals were detected. Septic shock was diagnosed according to Third International Consensus Definitions for Sepsis and Septic Shock [14].

### Microbiological methods

Blood samples (10 ml) from each patient were collected in aerobic and anaerobic culture bottles according to the standard procedure at Shanghai Tongji Hospital. Samples were loaded into the Bactec FX400 blood culture system (Becton Dickinson, Franklin Lakes, NJ, USA), and positive bottles were subcultured and examined by Gram stain. Isolates were identified to species level by matrix-assisted desorption/ionisation time-of-flight mass spectrometry (MALDI-TOF MS), performed using Microflex LT (Bruker Daltonics, Bremen, Germany), and antibiotic susceptibility was determined in the VITEK-2 compact system (bioMerieux, Marcy L'Etoile, France).

### Data collection

The following anonymous information was collected for each subject: age, sex, source department, comorbidity, drug resistance, TTP, antibiotic therapy, haemodialysis, transfusion, surgery, length of hospital stay and blood examination indices, including serum creatinine (SCR), total bilirubin (TBIL), mean arterial pressure (MAP), blood platelet count (PLT), procalcitonin (PCT), white blood cell count (WBC) and C-reactive protein (CRP).

### Statistical analyses

The median and interquartile range (M, P25–P75) or frequency and proportion (*N*, %) were used to describe the basic information of the enrolled subjects, and differences between groups were compared using the Mann–Whitney *U* test and *χ*^2^ or Fisher's exact probability test. A bar chart was drawn to map the distribution of TTP values, and a receiver operating characteristic (ROC) curve along with an area under the curve (AUC) analysis was developed to explore the predictive effect of TTP on in-hospital mortality. Youden's index was calculated to estimate the appropriate TTP cutoff value. Univariate logistic regression analyses were conducted on all variables and those with statistical significance were entered into multivariate logistic regression analyses. TTP values were included into all the latter analyses regardless of their statistical significance by univariate analyses, to explore correlations with in-hospital mortality, septic shock and ICU admission. Odds ratios (OR) and corresponding 95% confidence intervals (95% CI) were calculated, and a two-sided *P* value >0.05 was statistically significant. Data were analysed using SPSS 24.0 software (IBM Corporation, Armonk, NY, USA).

## Results

A total of 220 patients with *K. pneumoniae* BSIs were identified, of which 72 were excluded (63 from the ICU, three had a polymicrobial BSI, one was an outpatient and five with incomplete data); the remaining 148 patients were enrolled in the study.

Table 1 shows that the median age of the study cohort was 68 years; ranging from 18–96 years; 66.2% were male, 99.3% had at least one comorbidity and 66.2% had undergone antibiotic therapy before blood sample collection. The median length of hospital stay was 22.0 (12.0–42.8) days. Drug resistance was detected in 41.2% *K. pneumoniae* isolates, 30.4% CRKP and 10.8% MDRKP. Drug-resistant isolates were more commonly recovered in death cases (53.2% *vs.* 35.6%). Two patients (1.4%) had undergone haemodialysis, 65.5% had received a blood transfusion and 60.1% had recent surgical treatment. In total, 47 patients (31.8%) had died, 16 (10.8%) had septic shock and 28.6% required admission to the ICU.

**Table 1.** Clinical characteristics of survival and death groups among patients with *K. pneumoniae* BSI

Variables	Total (*N* = 148)		Survival (*N* = 101)		Death (*N* = 47)	
	*N*	M (P25–P75)/%	*N*	M (P25–P75)/%	*N*	M (P25–P75)/%
Age (years)	148	68.0 (60.0–79.0)	101	67.0 (56.0–75.5)	47	72.0 (63.0–83.0)
Gender						
Male	98	66.2	69	68.3	29	61.7
Female	50	33.8	32	31.7	18	38.3
Comorbidity						
No	1	0.7	1	1.0	0	0.0
Yes	147	99.3	100	99.0	47	100.0
SCR (μmol/l)	147	77.0 (57.0–114.0)	101	73.0 (55.5–96.5)	46	113.5 (67.0–229.8)
TBIL (μmol/l)	144	19.2 (11.5–35.5)	99	18.9 (11.6–29.9)	45	22.30 (11.1–56.6)
MAP (mmHg)	148	93.3 (85.3–97.3)	101	93.3 (86.7–97.3)	47	93.3 (74.7–100.0)
PLT(10^9^/l)	148	137.5 (62.3–209.3)	101	148.0 (80.5–236.0)	47	115.0 (13.0–177.0)
PCT (ng/ml)	140	2.1 (0.2–14.2)	96	1.3 (0.2–8.7)	44	8.6 (0.6–21.2)
WBC (10^9^/l)	148	10.8 (6.5–15.9)	101	10.9 (7.5–15.8)	47	10.3 (2.9–17.0)
CRP (mg/l)	148	124.0 (65.6–179.2)	101	105.4 (53.8–160.0)	47	160.0 (115.0–213.2)
Hospital stay (days)	148	22.0 (12.0–42.8)	101	22.0 (12.0–42.0)	47	21.0 (11.0–48.0)
TTP (h)	148	11.0 (7.7–16.1)	101	11.5 (8.6–21.3)	47	9.3 (6.6–13.1)
Drug resistance						
No	87	58.8	65	64.4	22	46.8
CRKP	45	30.4	28	27.7	17	36.2
MDRKP	16	10.8	8	7.9	8	17.0
Antibiotic therapy^a^						
No	50	33.8	40	39.6	10	21.3
Yes	98	66.2	61	60.4	37	78.7
Haemodialysis						
No	146	98.7	99	98.0	47	100.0
Yes	2	1.4	2	2.0	0	0.0
Transfusion						
No	51	34.5	43	42.6	8	17.0
Yes	97	65.5	58	57.4	39	83.0
Surgery						
No	59	39.9	44	43.6	15	31.9
Yes	89	60.1	57	56.4	32	68.1
ICU admission						
No	105	71.4	73	73.0	32	68.1
Yes	42	28.6	27	27.0	15	31.9
Septic shock						
No	129	89.0	95	96.9	34	72.3
Yes	16	11.0	3	3.1	13	27.7

Annotation: ^a^, antibiotic therapy before blood collection.

**Fig. 1.**
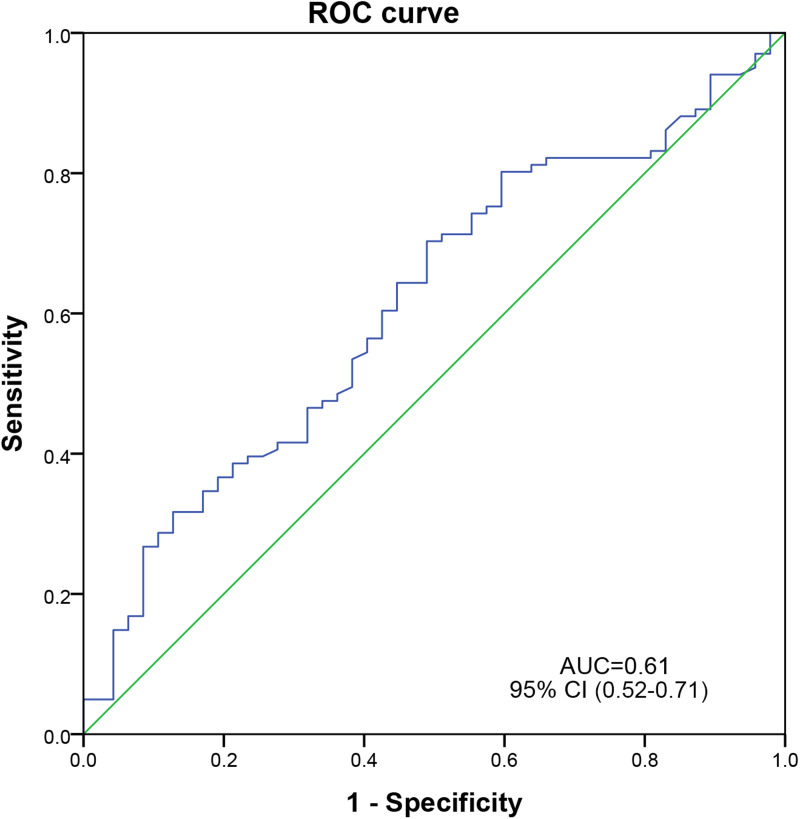
ROC curve of TTP on in-hospital mortality among patients with *K. pneumoniae* BIS.

The median TTP for all patients was 11.0 (7.7–16.1 h and values ranged from 1.2–105.5 h), 11.5 (8.6–21.3 h) for the survival group and 9.3 (6.6–13.1 h) in the death group (Supplementary Fig. S1). Figure 1 shows that the ROC curve of TTP discriminated in-hospital mortality among patients with *K. pneumoniae* BSI, with an AUC of 0.61 (95% CI 0.52–0.71). A maximum Youden's index was reached when the TTP was 9.4 h, with a sensitivity of 70.3% and specificity of 51.1%. An early TTP (<9.4 h) was observed in 36.5% of the cohort patients who had higher in-hospital mortality rates than those with late TTP (44.4% *vs.* 24.5%). Among patients without antibiotic treatment before blood culture, early TTP was associated with in-hospital mortality according to *χ*^2^ tests after correlation for continuity (*P* = 0.02). The ROC showed a predictive, but statistically nonsignificant value of TTP (AUC = 0.67, *P* = 0.49). By contrast, statistically significant differences in TBIL, PLT, WBC, transfusion, surgery, and in-hospital mortality rates were observed between early (<9.4 h) and late TTP (≥9.4 h) patients with *K. pneumoniae* BSI (Supplementary Table S1).

In the univariate logistic regression analysis of factors related to in-hospital mortality of the study patients, several factors were identified (Table 2) including: age > 60 years (OR = 3.6, 95% CI 1.4–9.4, *P* < 0.01), SCR > 106 μmol/l (OR = 4.6, 95% CI 2.1–10.0, *P* < 0.01), PCT > 2 ng/ml (OR = 2.5, 95% CI 1.2–5.2, *P* = 0.02), WBC < 4 × 10^9^/l or >20 × 10^9^/l (OR = 3.7, 95% CI 1.7–7.7, *P* < 0.01), CRP > 100 mg/l (OR = 3.5, 95% CI 1.6–7.8, *P* < 0.01), early TTP (OR = 2.5, 95% CI 1.2–5.0, *P* = 0.01), antibiotic therapy before blood collection (OR = 2.4, 95% CI 1.1–5.4, *P* = 0.03) and transfusion (OR = 3.6, 95% CI 1.5–8.5, *P* < 0.01). However, in the multivariate analysis, only age > 60 years (OR = 3.6, 95% CI 1.0–12.2, *P* = 0.04), SCR>106 μmol/l (OR = 6.1, 95% CI 2.0–18.4, *P* < 0.01), WBC<4 × 10^9^/l or >20 × 10^9^/l (OR = 4.4, 95% CI 1.5–12.4, *P* < 0.01), and CRP > 100 mg/l (OR = 4.0, 95% CI 1.3–12.0, *P* = 0.01) were confirmed as independent risk factors. Likewise, no significant influence of early TTP on mortality (OR = 2.7, 95% CI 1.0–7.4, *P* = 0.06) was observed.

**Table 2.** Factors related to in-hospital mortality among patients with *K. pneumoniae* BSI: univariate and multivariate logistic regression

Variables	Univariate logistic regression			Multivariate logistic regression		
	OR	95% CI	*P*	OR	95% CI	*P*
Age > 60 years	3.6	1.4–9.4	<0.01	3.6	1.0–12.2	0.04
Female	1.3	0.7–2.8	0.43			
SCR > 106 μmol/l	4.6	2.1–10.0	<0.01	6.1	2.0–18.4	<0.01
TBIL > 22.24 μmol/l	1.8	0.9–3.7	0.10	1.4	0.5–3.8	0.49
MAP > 60 mmHg	0.4	0.1–1.9	0.27			
PLT < 100/>300^10^9^/l	1.1	0.6–2.3	0.70			
PCT > 2 ng/ml	2.5	1.2–5.2	0.02	1.5	0.5–4.5	0.43
WBC < 4/>20^10^9^/l	3.7	1.7–7.7	<0.01	4.4	1.5–12.4	<0.01
CRP > 100 mg/l	3.5	1.6–7.8	<0.01	4.0	1.3–12.0	0.01
Hospital stay (days)	1.0	1.0–1.0	0.50			
Early TTP	2.5	1.2–5.0	0.01	2.7	1.0–7.4	0.06
Drug resistance						
No	1.0			1.0		
CRKP	1.8	0.8–3.9	0.14	1.4	0.4–4.6	0.55
MDRKP	3.0	1.0–8.8	0.05	1.0	0.2–4.5	0.99
Antibiotic therapy^a^	2.4	1.1–5.4	0.03	2.4	0.7–8.1	0.16
Transfusion	3.6	1.5–8.5	<0.01	2.6	0.8–8.4	0.11
Surgery	1.6	0.8–3.4	0.18			

Annotation: *N* = 135; Hosmer–Lemeshow test: *P* = 0.51; Overall prediction accuracy: 78.5%; ^a^, antibiotic therapy before blood collection.

In the univariate logistic regression analysis of factors related to septic shock in 16 patients (Table 3), PCT > 2 ng/ml (OR = 4.6, 95% CI 1.2–17.0, *P* = 0.02), WBC < 4 × 10^9^/l or >20 × 10^9^/l (OR = 3.7, 95% CI 1.3–10.8, *P* = 0.02), MDRKP (OR = 5.3, 95% CI 1.2–22.4, *P* = 0.03), antibiotic therapy before blood collection (OR = 8.6, 95% CI 1.1–67.2, *P* = 0.04), and transfusion (OR = 8.6, 95% CI 1.1–67.2, *P* = 0.04) proved to be significant risk factors.

**Table 3.** Factors related to septic shock among patients with *K. pneumoniae* BSI: univariate logistic regression

Variables	Univariate logistic regression		
	OR	95% CI	*P*
Age > 60 years	1.7	0.5–6.5	0.41
Female	0.9	0.3–2.7	0.82
SCR > 106 μmol/l	1.2	0.4–3.6	0.79
TBIL > 22.24 μmol/l	1.1	0.4–3.1	0.87
MAP > 60 mmHg	0.9	0.1–7.5	0.89
PLT < 100/>300^10^9^/l	2.1	0.7–6.1	0.17
PCT > 2 ng/ml	4.6	1.2–17.0	0.02
WBC < 4/>20^10^9^/l	3.7	1.3–10.8	0.02
CRP > 100 mg/l	2.2	0.7–7.3	0.18
Hospital stay (days)	1.0	1.0–1.0	0.63
Early TTP	1.8	0.6–5.1	0.27
Drug resistance			
No	1.0		
CRKP	2.9	0.9–9.8	0.08
MDRKP	5.3	1.2–22.4	0.03
Antibiotic therapy^a^	8.6	1.1–67.2	0.04
Transfusion	8.6	1.1–67.2	0.04
Surgery	3.0	0.8–11.1	0.10

Annotation: *N* = 137; ^a^, antibiotic therapy before blood collection.

Several factors related to ICU admission were identified in the univariate analysis (Table 4) including: gender, elevations of MAP and hospital stays, CRKP, MDRKP, antibiotic therapy before blood collection, transfusion and surgery; by multivariate analysis, only surgery (OR = 3.6, 95% CI 1.2–11.1, *P* = 0.03) was linked to ICU admission. Female sex (OR = 0.2, 95% CI 0.1–0.6, *P* < 0.01) was an independent protective factor. No significant influence of increasing TTP on ICU admission was found in the univariate (OR = 1.0, 95% CI 1.0–1.0, *P* = 0.07) or multivariate analyses (OR = 1.0, 95% CI 1.0–1.0, *P* = 0.32).

**Table 4.** Factors related to ICU admission among patients with *K. pneumoniae* BSI: univariate logistic regression and multivariate logistic regression

Variables	Univariate logistic regression			Multivariate logistic regression		
	OR	95% CI	*P*	OR	95% CI	*P*
Age > 60 years	1.9	0.8–4.7	0.14			
Female	0.3	0.1–0.7	<0.01	0.2	0.1–0.6	<0.01
SCR (μmol/l)	1.0	1.0–1.0	0.74			
TBIL (μmol/l)	1.0	1.0–1.0	0.23			
MAP (mmHg)	1.0	1.0–1.0	0.04	1.0	1.0–1.0	0.28
PLT (10^9^/l)	1.0	1.0–1.0	0.06	1.0	1.0–1.0	0.19
PCT (ng/ml)	1.0	1.0–1.0	0.47			
WBC (10^9^/l)	1.0	1.0–1.1	0.26			
CRP (mg/l)	1.0	1.0–1.0	0.81			
Hospital stay (days)	1.0	1.0–1.0	<0.01	1.0	1.0–1.0	0.24
TTP (h)	1.0	1.0–1.0	0.07	1.0	1.0–1.0	0.32
Drug resistance						
No	1.0			1.0		
CRKP	4.6	2.0–10.3	<0.01	1.8	0.6–5.0	0.28
MDRKP	4.6	1.4–14.6	0.01	3.1	0.7–13.4	0.13
Antibiotic therapy^a^	10.5	3.1–36.2	<0.01	4.1	1.0–17.7	0.05
Transfusion	2.8	1.2–6.7	0.02	1.6	0.5–5.3	0.43
Surgery	5.9	2.3–15.1	<0.01	3.6	1.2–11.1	0.03

Annotation: *N* = 147; Hosmer–Lemeshow test: *P* = 0.40; Overall prediction accuracy: 71.4%; ^a^, antibiotic therapy before blood collection.

## Discussion

In the present study, the median TTP was 11.0 h for patients with *K. pneumoniae* BSI, and there was a significant discriminative effect of its value on in-hospital mortality, with an optimal cut-off of 9.4 h according to the ROC curve. This result was similar to the median TTP of 10 h observed in a recent study on newborns [15] and was lower than the median of 14.2 h and cut-off value of 13 h among paediatric patients [13], but higher than median and cut-off values (9.6 and 7 h respectively) for *K. pneumoniae* BSI in adults in another study [12]. These outcomes can perhaps be partly explained by differences in physiological properties and bacterial load between children and adults. Unlike the latter two studies, antibiotic therapy was administered to over half of our patients in both the survival and mortality groups representing a much higher comparative number [12, 13], which may account for such differences [16]. Furthermore, it is inaccurate to divide subjects into short and long TTP (break point = 7 h) groups based only a higher OR without taking account of the sensitivity and specificity of the blood culture system [12].

In our series approximately one-third (36.5%) of patients exhibited an early TTP (<9.4 h); these patients had a 2.5 times higher risk of in-hospital mortality than those with late TTP values, according to univariate analysis. This observation was consistent with previous *K. pneumoniae* BSI studies [12, 13]. Among patients who had not received antibiotics prior to blood culture, early TTP was associated with in-hospital mortality despite a lack of significant discrimination in ROC (Supplementary Fig. S2). After controlling for several patient characteristics (Tables 1 and 2) patients with an early TTP had a 2.7 times higher risk of in-hospital death, but this did not prove statistically significant by multivariate analysis. Similar results were reported for BSI due to *Enterococcus* spp. [17]. Here, we observed a number of independent risk factors for in-hospital mortality which included age > 60 years, SCR > 106 μmol/l, abnormal WBC count, and CRP > 100 mg/l. SCR is a biomarker for renal function and high values are indicative of acute kidney injury and associated with mortality of hospitalised patients [18]. Likewise, the WBC count is an initial diagnostic marker of sepsis [19] and correlates to severe bacteraemia [20]. Moreover, as found in this study, an elevated CRP value was reported to be linked to mortality in *S. aureus* bacteraemia [10].

Neither early TTP nor increasing TTP proved to be significant factors in septic shock or ICU admission for patients with *K. pneumoniae* BSI. However, a meta-analysis of the literature noted that a short TTP was a general predictor of septic shock in patients with general bacterial BSI [21], but did not specifically include *K. pneumoniae*. Paediatric patients with early TTP were reported to be more likely to present with septic shock [13], was not found here which might suggest that the prognosis of young patients is less affected by underlying conditions more commonly associated with the elderly.

This study has a number of limitations mainly due to its retrospective and observational nature. Consequently, diagnostic clinical score data, sources and sites of infection along with underlying conditions and comorbidities were not recorded. Likewise, the relatively low sample size of the cohort may have impacted on the validity of the multivariate analysis.

In conclusion, our findings indicate the relatively high in-hospital fatality rate of patients with *K. pneumoniae* BSI was affected by age and blood markers, notably SCR, CRP and WBC. Early TTP was statistically associated with in-hospital mortality of the patient cohort but a higher risk of fatality was not evident for septic shock or ICU admission among the early TTP group.

## Data Availability

The data that support the findings of this study are available on request from the corresponding author. The data are not publicly available due to their containing information that could compromise the privacy of research participants.

## Supplementary material

For supplementary material accompanying this paper visit http://doi.org/10.1017/S0950268823000262. click here to view supplementary material
