# Supplementary material for: Is early time to positivity of blood culture associated with clinical prognosis in patients with Klebsiella pneumoniae bloodstream infection?
Source: Epidemiol Infect. 2023 Feb 21;151:e43. doi: 10.1017/S0950268823000262 (PMC10028975; doi:10.1017/S0950268823000262)
Supplement: Supplementary file 1 [file S0950268823000262sup001.docx]

Epidemiology and Infection

**Is Early Time to Positivity of Blood Culture Associated with Clinical Prognosis in Patients with *Klebsiella pneumoniae* Bloodstream Infection?**

Weiwei Hou^a, 1^, Tiantian Han^b, 1^, Guangbo Qu^c^, Yehuan Sun^c, d, e^, Dianyu Yang^a^, Yan Lin^b, *^

Supplementary Material


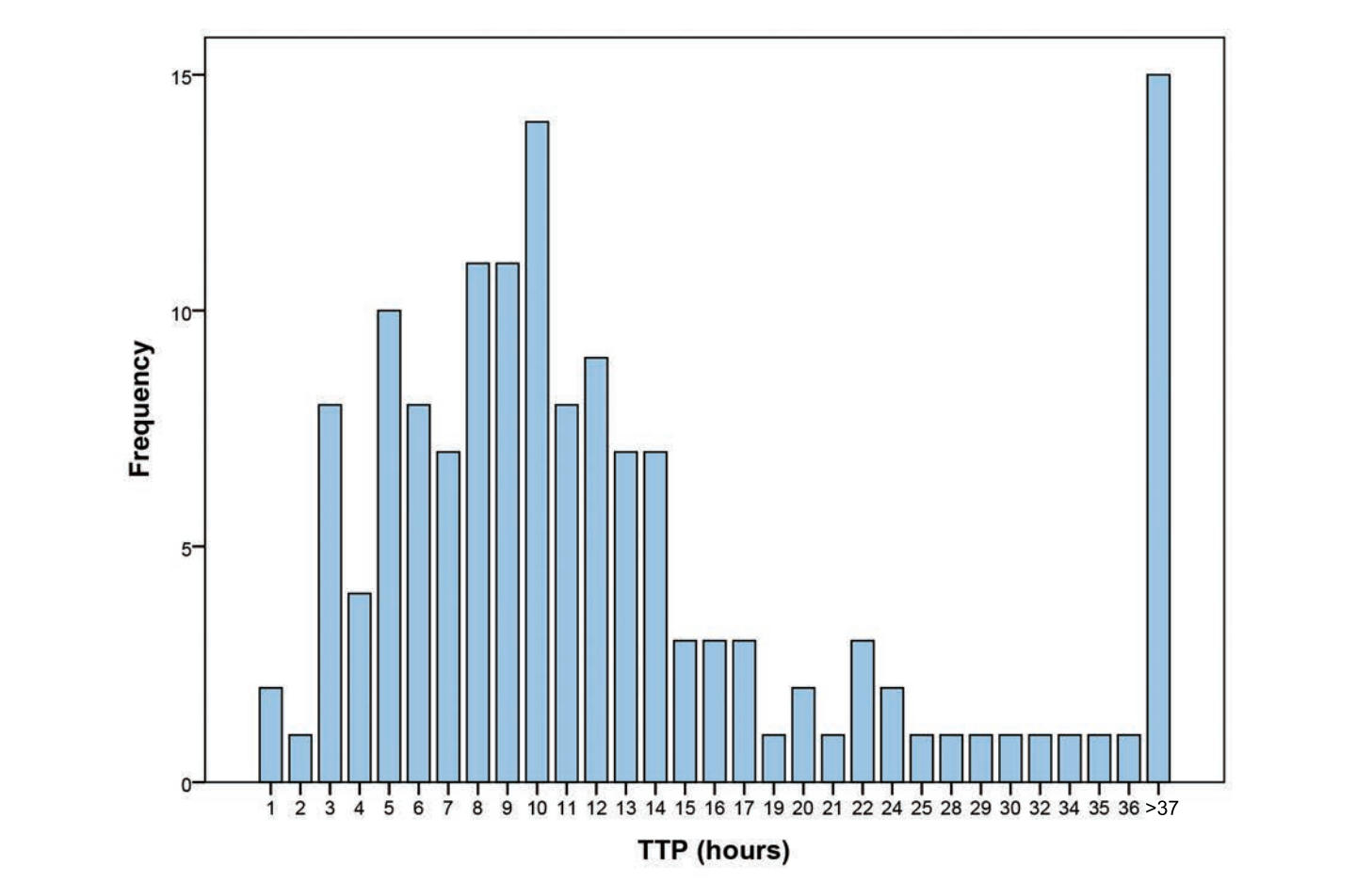
Figure S1. Distribution of TTP among patients with K. pneumoniae BSI.


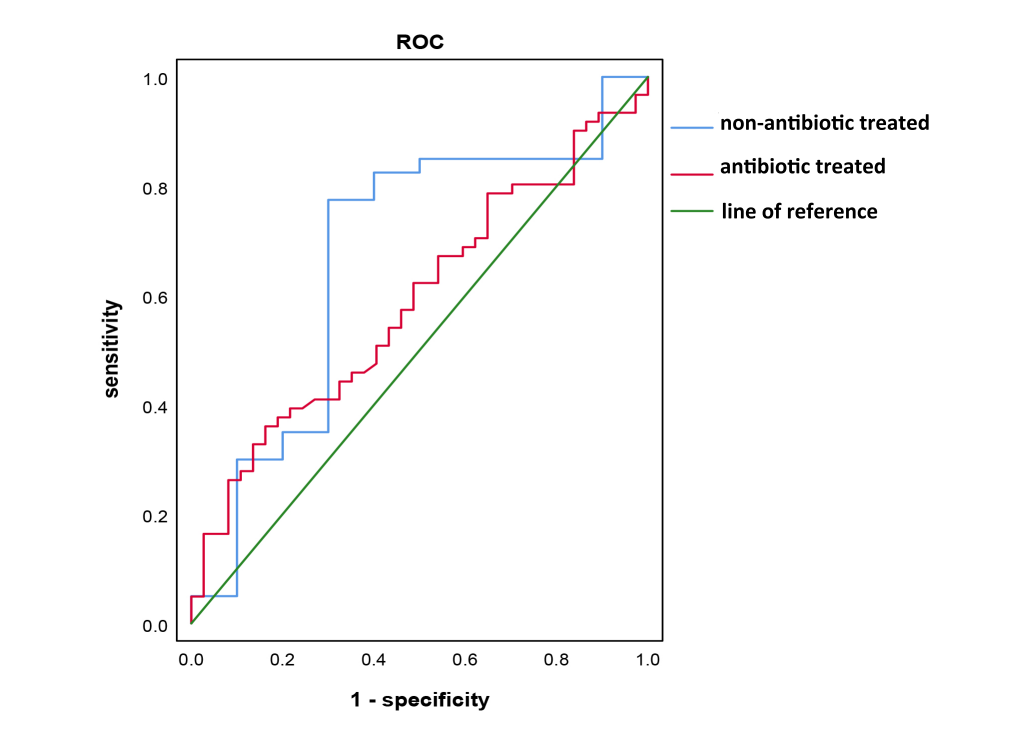
Figure S2. ROC curve of TTP on in-hospital mortality among non-antibiotic and antibiotic treated patients.

Table S1. Comparison of clinical characteristics between early TTP and late TTP groups among patients with *K. pneumoniae* BSI.

| Variables | TTP≥9.4h (N=94) | | TTP<9.4h (N=54) | | *p* |
| --- | --- | --- | --- | --- | --- |
|  | N | M(P25-P75) / % | N | M(P25-P75) / % |  |
| Age (years) | 94 | 69.5 (55.8-79.0) | 54 | 67.0 (60.0-80.5) | 0.82 |
| Gender |  |  |  |  | 0.53 |
| Male | 64 | 68.1 | 34 | 63.0 |  |
| Female | 30 | 31.9 | 20 | 37.0 |  |
| Comorbidity | 93 | 98.9 | 54 | 100.0 | 1.00* |
| SCR (μmol/L) | 93 | 79.0 (57.5-109.5) | 54 | 75.5 (56.0-140.0) | 0.78 |
| TBIL (μmol/L) | 92 | 17.3 (10.4-29.1) | 52 | 22.80 (14.0-55.5) | 0.02 |
| MAP (mmHg) | 94 | 93.3 (87.2-99.8) | 54 | 93.3 (83.3-96.8) | 0.22 |
| PLT(10^9^/L) | 94 | 160.5 (96.5-236.0) | 54 | 88.5 (19.3-172.5) | <0.01 |
| PCT (ng/ml) | 91 | 2.2 (0.3-14.6) | 49 | 1.4 (0.2-14.0) | 0.57 |
| WBC (10^9^/L) | 94 | 12.4 (9.2-16.5) | 54 | 7. 9 (3.0-13.1) | <0.01 |
| CRP (mg/L) | 94 | 129.4 (65.8-182.5) | 54 | 117.6 (57.3-168.0) | 0.53 |
| Hospital stay (d) | 94 | 22.0 (12.8-42.0) | 54 | 21.5 (10.0-48.8) | 0.77 |
| Drug resistance |  |  |  |  | 0.66 |
| No | 53 | 56.4 | 34 | 63.0 |  |
| CRKP | 31 | 33.0 | 14 | 25.9 |  |
| MDRKP | 10 | 10.6 | 6 | 11.1 |  |
| Antibiotic therapy^1^ | 61 | 64.9 | 37 | 68.5 | 0.65 |
| Hemodialysis | 2 | 2.1 | 0 | 0.0 | 0.53* |
| Transfusion | 55 | 58.5 | 42 | 77.8 | 0.02 |
| Surgery | 50 | 53.2 | 39 | 72.2 | 0.02 |
| ICU admission | 26 | 28.0 | 16 | 29.6 | 0.26 |
| Septic shock | 8 | 8.8 | 8 | 14.8 | 0.26 |
| Death | 23 | 24.5 | 24 | 44.4 | 0.01 |

Annotation: *, Fisher’s exact probability; ^1^, antibiotic therapy before blood collection.
